# Combining mussel with fucoidan as a supplement for joint pain and prediabetes: Study protocol for a randomized, double-blinded, placebo-controlled trial

**DOI:** 10.3389/fnut.2022.1000510

**Published:** 2022-09-21

**Authors:** Audrey Tay, Yannan Jiang, Nada Signal, Daniel O'Brien, Jinsong Chen, Rinki Murphy, Jun Lu

**Affiliations:** ^1^Faculty of Medical and Health Sciences, School of Medicine, University of Auckland, Auckland, New Zealand; ^2^Auckland Diabetes Centre, Auckland District Health Board, Auckland, New Zealand; ^3^Department of Statistics, Faculty of Science, University of Auckland, Auckland, New Zealand; ^4^Faculty of Health and Environmental Sciences, School of Clinical Sciences, Auckland University of Technology, Auckland, New Zealand; ^5^Faculty of Medical and Health Sciences, School of Population Health, University of Auckland, Auckland, New Zealand; ^6^Department of Whitiora Diabetes, Counties Manukau District Health Board, Auckland, New Zealand; ^7^Maurice Wilkins Centre for Biodiscovery, Auckland, New Zealand; ^8^Faculty of Health and Environmental Sciences, School of Sciences, Auckland University of Technology, Auckland, New Zealand; ^9^Institute of Biomedical Technology, Auckland University of Technology, Auckland, New Zealand; ^10^College of Food Science and Technology, Nanchang University, Nanchang, China; ^11^College of Food Engineering and Nutrition Sciences, Shaanxi Normal University, Xi'an, China; ^12^School of Perfume and Aroma Technology, Shanghai Institute of Technology, Shanghai, China

**Keywords:** prediabetes, joint pain, fucoidan, green-lipped mussels, randomized control trial (RCT), Chinese

## Abstract

**Introduction:**

Pharmaceutical drugs are beneficial to inflammatory conditions but with side effects, which led to the search for alternative therapies. *Perna canaliculus*, the New Zealand green-lipped mussel, have shown promise in placebo-controlled trials for inflammatory conditions. Fucoidan, an extract from seaweed *Undaria pinnatifida*, has been found to have beneficial effects on joint pain and insulin resistance. However, green-lipped mussel and fucoidan have never been combined.

**Methods and analysis:**

A parallel, two-arm, double-blind, randomized, placebo-controlled trial will be conducted in New Zealand to determine whether a food product supplemented with green-lipped mussel and fucoidan improves joint pain and/or insulin resistance. Those who are ethnically Chinese, are aged over 30 years, have prediabetes and hip or knee joint pain will be eligible to participate. They will be randomized at 1:1 ratio to consume either dark chocolate supplemented with 1000 mg mussel powder and 1000 mg fucoidan or dark chocolate with no active substances daily for 100 days. The primary endpoints are change in insulin resistance and patient-reported joint pain. Secondary endpoints include anthropometry, fasting glucose and insulin, HbA1c, inflammatory markers, satiety, quality of life, physical function, pain intensity, and analgesic medication use. A sample size of 150 (75 per arm) will provide 90% power at an overall significance level of 5% (two-sided) to detect a standardized effect size of 0.625 on either of the two co-primary outcomes allowing for 10% loss.

**Ethics and dissemination:**

The study was approved by the Health and Disability Ethics Committee (number: 20/STH/153). Results will be made available to participants, funders, and other researchers.

**Discussion:**

This trial will provide data on the potential utility of a mussel-fucoidan supplement in reducing joint pain and/or insulin resistance, to inform the development of a supplemented food product suitable for the Chinese market.

**Clinical trial registration:**

https://trialsearch.who.int/Trial2.aspx?TrialID=ACTRN12621000413820, ANZCTR Registration: ACTRN12621000413820, on 15 April 2021.

## Introduction

Inflammatory conditions, such as chronic musculoskeletal joint pain and insulin resistance, strongly contribute to the global burden of disease. Osteoarthritis and other causes of chronic musculoskeletal joint pain combined leads to an estimated 21.3% of the total years lived with disability globally (1). Chronic joint pain also impacts an individual's quality of life, participation in work and social activities, and economic independence (1). Type 2 diabetes, characterized by insulin resistance, is also a global health issue with prevalence rates rising (2). Diabetes is a major cause of blindness, kidney failure, cardiovascular disease, and lower-limb amputations (2). Therefore, preventative measures to avoid diabetes, joint pain, and their complications is a global challenge. Although pharmaceutical drugs for both chronic joint pain and diabetes are beneficial, they also have considerable costs and experienced side effects (3). Investigating potential alternatives to these pharmacological therapies is warranted.

One potential complementary therapy stems from *Perna canaliculus*, the New Zealand green-lipped mussel, which is native to the New Zealand coast. Nutritional supplements for inflammatory conditions, such as osteoarthritis, which contain *P. canaliculus* extracts, have shown promising results in placebo-controlled trials (4, 5). A six-month study evaluated the effect of Lyprinol^®^, a lipid extract of *P. canaliculus*, among 80 Chinese patients with knee osteoarthritis (6). There was a significantly greater reduction in perceived pain, and improvement in patients' global assessment of their arthritis, among those who took Lyprinol^®^ when compared with controls. Thus, the potential use of *P. canaliculus* for joint pain should be explored further.

A waste product of the mussel harvest process is *Undaria pinnatifida*, a species of New Zealand seaweed that infests the lines of mussel farms and interferes with the mussel harvest machinery (7). However, this seaweed species may also provide another potential complementary therapy. Polysaccharides called fucoidans can be extracted from *U. pinnatifida* and have been found to have various bioactive properties in *in vitro* models (8–11). Fucoidan extracts from other algal species have been shown to have anti-inflammatory effects and decrease the symptoms of osteoarthritis among a small sample of adults with knee osteoarthritis (12). In an animal study, it has been shown to inhibit carrageenan-induced oedema and ameliorated mechanical allodynia (13). *In vitro* studies also suggest that fucoidan may have preferential inhibition effect on cyclooxygenase-2, hence exhibit anti-inflammatory property (14). Therefore, it has the potential to be used for joint pain.

Fucoidan extract from *U. pinnatifida* has also been found to inhibit starch hydrolysing enzymes, such as α-amylase and α-glucosidase (8), which can slow down the digestion of carbohydrates. It may also improve insulin-stimulated glucose uptake and inhibit basal lipolysis in adipocytes without inducing adipogenesis, as previously shown using an *in vitro* model (15). Fucoidan mixed with traditional Chinese medicine has also demonstrated protective effects in diabetic rats, suggesting possible use for the treatment of diabetes mellitus (16). A recent clinical trial conducted in Australia (*n* = 72) investigated whether fucoidan extract reduced insulin resistance in an obese, non-diabetic population (17). Participants took capsules of either fucoidan/polyphenol extract (500 mg from *Fucous vesiculosus*) or a placebo. After 90-days of twice-daily supplementation, insulin resistance remained unchanged, as calculated using the homeostasis model of assessment (HOMA) score. However, participants had normal glucose tolerance (18). Recruiting individuals with established insulin resistance, such as prediabetes, may have illustrated more clinically meaningful effects.

Products containing fucoidan extracts have attained regulatory approvals in certain countries for use in foods and dietary supplements (19). The Food and Drug Administration in the United States of America (USA) permits consumption of fucoidan extracts from *U. pinnatifida* and *F. vesiculosus* at levels of up to 250 mg per day. Although, recent studies have used larger doses and reported no signs of clinical toxicity (12, 20, 21). Fucoidan extracts have particularly gained interest in China, such as the Haikun Shenxi Capsule of which the main active component is fucoidan, which has been approved for treating renal diseases (22).

Combining green-lipped mussel and fucoidan extracts is a novel innovation and may produce additive benefits. To our knowledge, this is the first study to test the anti-diabetic and joint pain effects of this combination. Therefore, this trial aims to gain new knowledge into the bioactivity of a mussel-fucoidan combination, particularly focusing on its potential effects on inflammation and glycaemic control. The results of this trial will help us to better understand the use and effectiveness of mussel-fucoidan health products among the Chinese population and will help inform product development for the Chinese market.

## Methods/design

### Trial aim and hypothesis

The primary objective of this trial is to determine whether a food product supplemented with green-lipped mussel and fucoidan powder improves joint pain and/or insulin resistance. We hypothesize that the mussel-fucoidan combination will be superior to placebo in reducing patient-reported joint pain and/or improving insulin resistance.

### Trial design

**Figure 1:**
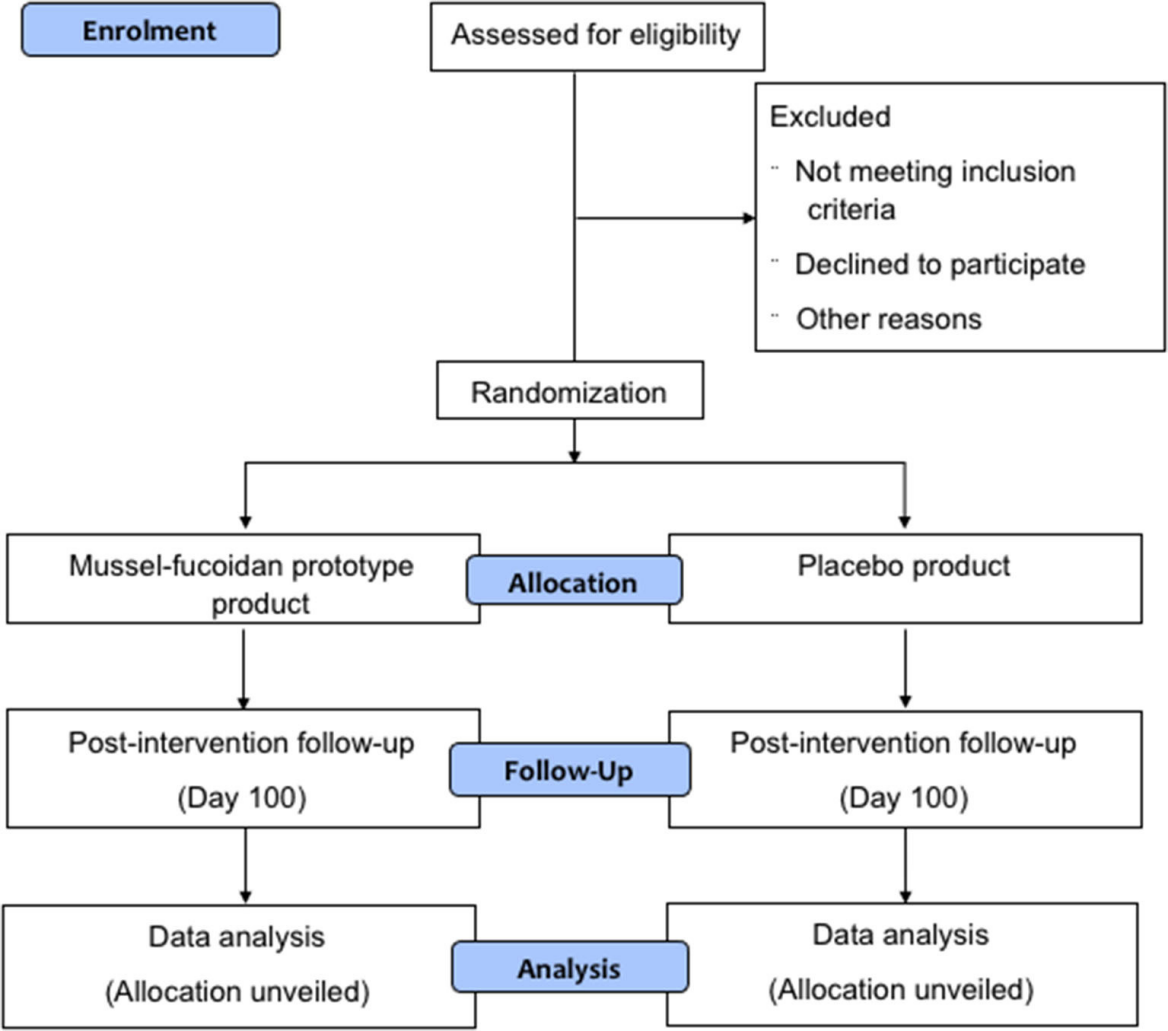
CONSORT diagram.

A parallel, two-arm, double-blind, randomized, placebo-controlled trial will be conducted in Auckland, New Zealand. The trial steering committee is located at the Auckland University of Technology (AUT) North Shore campus. Ethical approval was granted by the Health and Disability Ethics Committee (HDEC; reference number: 20/STH/153). The protocol was prepared following Standard Protocol Items: Recommendations for Interventional Trials (SPIRIT) statement (**Additional File 1**). The CONSORT flow diagram is present in Figure 1. The funding application including the trial design has been reviewed by the funding agency the New Zealand National Science Challenge – High Value Nutrition.

### Patient and public involvement

We invited eight people who met the proposed eligibility criteria to participate and provide feedback on the trial design *via* telephone interviews. They were generally supportive of the trial; feedback centered on the proposed recruitment methods, the trial schedule of assessments and data collection, and methods for disseminating results. Engaging with this group supported the refinement of the trial design to ensure that the study protocol is feasible and that the outcomes are valuable to key stakeholders.

### Participant characteristics

Participants will be recruited through primary healthcare organizations, general practices, Asian health organizations, and advertising in Chinese media. Adults who are ethnically Chinese and live in Auckland, New Zealand, will be invited to participate in this trial. The eligibility criteria are presented in Table 1.

**Table 1:** Trial eligibility criteria.

**Inclusion criteria (all)**	**Exclusion criteria (any of the below)**
• Of Chinese ethnicity (both parents are ethnically Chinese) • Lives in Auckland • Aged over 30 years • Able to speak and understand English and/or Mandarin • HbA1c between 41 and 49 mmol/mol at screening • Ongoing knee or hip joint pain for the last 3 months or longer (i.e. diagnosed with osteoarthritis) • Owns a smartphone or tablet, has access to an Internet connection, and is willing to use the WeChat app	• Diabetes (type 1 or 2) • Known or suspected seafood (fish and shellfish) allergy • Currently taking a green-lipped mussel supplement • Previous joint replacement surgery, or planning this within the next six months • Pregnant or breastfeeding, or intending to become pregnant within the next 6 months • Previous bariatric surgery • Conditions that may influence body weight regulation (e.g. malabsorption, thyroid disorders) • Taking any glucose-lowering medications, systemic steroids, or blood thinners • History of thalassemia, asthma, gout, liver disease, kidney disease • Planning major changes to physical activity within the next 6 months • Significant weight loss or weight gain within the last 6 months • Fear of needles or giving blood • No access to a smartphone, tablet, or Internet

### Informed consent

All participants will be screened for eligibility based on the outlined criteria. Participants will be encouraged to discuss their involvement in this trial with others and ask the research team any questions before agreeing to participate. The principal investigator (PI) will ensure all participants provide written informed consent before data collection. All participant-facing documents will be available in both English and Mandarin. During data collection, research assistants fluent in both English and Mandarin will be present to support the participants' engagement in the research process. Participants can withdraw at any time.

### Preparation of the mussel-fucoidan test food and placebo

Mussel powder was supplied by Cfarmx New Zealand Limited (Auckland, New Zealand). The powder contains around 5% moisture, 12% lipid, 55% crude protein, 22% ions (ash) and 5% carbohydrate. The omega-3 lipid content is more than 2%. New Zealand green shell mussels are sustainably farmed with little environmental impact and are rated by the Monterey Bay Seafood Watch Programme as one of the most sustainable seafood products in the world. Seaweed sporophylls have been purchased from a New Zealand supplier and processed by NZ Extract Limited (New Zealand), Callaghan Innovation (New Zealand), and Alaron Products Limited (New Zealand) to produce 25 kg of fucoidan. The fucoidan product contains around 70% carbohydrate, 28% ions (ash) and 1% protein. The fucose and xylose content is more than 20% each, the sulphation level is around 25%, and the average molecular weight is around 330 kDa. The fucoidan produced was used to formulate a mussel-fucoidan food product in the food laboratory of AUT in Auckland, New Zealand. The food product has been tested for flavor and consumer acceptability. Alaron Products Limited manufactured the mussel-fucoidan prototype food product according to our formulation. Beyond Capital MedTech Management Limited has acquired the necessary food product certifications for the batch of fucoidan used, as well as the prototype food product.

### Randomization and concealment

Eligible participants will be randomly assigned to receive either the mussel-fucoidan prototype product or the placebo in a 1:1 ratio. The trial biostatistician will prepare the blinded randomization lists using permutated block randomization with variable block sizes. Random pack numbers will be generated in the drug labeling list and pre-packed in identical boxes by Alaron Products Limited. Once a participant has provided informed consent and has been confirmed eligible to be randomized, the database will show the pack number linked to the participant in the allocated group. A research assistant will provide participants with their correct treatment packs, with no information on actual group allocation. Neither the participant nor any research team member will know the treatment product indicated by the randomization. During the trial, the blind may be broken in emergencies, such as when knowledge of the participant's treatment group is necessary for further participant management.

### Interventions

All participants will receive either the treatment intervention or a placebo for 100 days. The mussel-fucoidan prototype product will be 20 g of dark chocolate containing 1000 mg of mussel powder and 1000 mg of fucoidan extract. The placebo will be 20 g of dark chocolate, without any active substances. Each dose will be individually and identically packaged. The prototype product and placebo have been determined as indistinguishable by sensory panelists. Participants will be encouraged to eat *ad libitum* over the 100-day period but will be advised of the caloric value of the dark chocolate should they wish to substitute foods with an equivalent caloric value. Participants who miss one dose will be encouraged to take two doses the following day to a maximum of two doses per day. The risk of overdosing is minimal as both fucoidan and mussel are food product with a GRAS (generally regarded as safe) status in FDA category.

### Outcome measures

**Figure 2:**
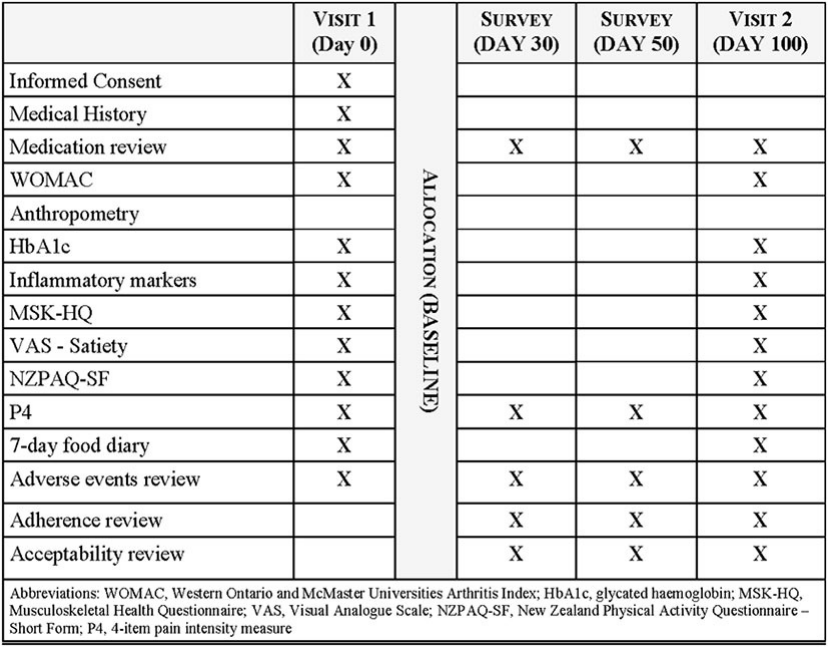
Trial schedule of assessments and data collection.

The primary endpoints are change in insulin resistance, defined by the HOMA values, and patient-reported pain measured by the Western Ontario and McMaster Universities Arthritis Index (WOMAC). Secondary endpoints include anthropometry, fasting glucose and insulin, glycated hemoglobin (HbA1c), inflammatory markers (C-reactive protein, tumor necrosis factor-alpha (TNF–α), interleukin (IL)-6, IL-2, IL-8, IL-1β, IL-10 and IL-4), physical function, quality of life, pain intensity, satiety on a VAS (0 = extremely hungry, 10 = extremely full), analgesic medication use, and parameters related to COX-2 inhibition. A 7-day food record and physical activity questionnaire will be collected pre- and post-intervention to determine any changes which may have influenced observed outcomes. The trial schedule of assessments and data collection is presented in Figure 2.

### Homeostasis model of assessment

The HOMA model is used to estimate insulin sensitivity using fasting plasma glucose and insulin concentrations (23). The liver and pancreatic β-cells function in a negative feedback loop, reflecting the balance between hepatic glucose output and insulin secretion. HOMA can be used to demonstrate the effects of an intervention on insulin resistance (24). The equation used to measure insulin resistance is (Fasting plasma insulin [mU/l) × Fasting plasma glucose (mmol/l)] ÷ 22.5.

### Perceived function and quality of life

The WOMAC is widely used to evaluate hip and knee osteoarthritis (25). It is a self-administered questionnaire that has been validated in both paper and electronic formats (26). The index consists of 24 items, each with a 5-point Likert scale, and is divided into three subscales: pain (five items), stiffness (two items), and physical function (17 items). Both the English and Chinese versions will be used in this study. The Chinese version of the WOMAC has been validated in previous studies (27, 28). The Musculoskeletal Health Questionnaire (MSK-HQ) contains 14 items and will be used at baseline and at Day 100 to measure pain severity, physical function, and quality of life (29). Pain will also be measured on Days 0, 30, 50, and 100 using the 4-item pain intensity measure (P4), which measures pain intensity over the past 2 days (30).

### Clinical data

Height will be measured using a calibrated stadiometer (SECA, USA) to the nearest 0.1 cm, without shoes. Weight will be measured using a calibrated electronic scale (SECA, USA) to the nearest 0.1 kg. Waist and hip circumferences will be measured over light clothing using a tape measure (Lufkin, USA) to the nearest 0.1 cm. Blood pressure will be measured using an automatic blood pressure monitor (OMRON, Japan) and an appropriately sized cuff while seated after 10 min of rest.

### Dietary and physical activity habits

Dietary habits will be assessed before and after the 100-day intervention using a 7-day food diary. Participants will be instructed to record details of all foods and drinks consumed over the seven days. Diaries will be entered into FoodWorks 10 Professional (Xyris Pty Limited, Australia) for analysis. Self-reported appetite will be measured pre- and post-intervention using a VAS questionnaire. Questions will focus on hunger, fullness, satiety, desire to eat, and prospective consumption (31). The New Zealand Physical Activity Questionnaire Short Form (NZPAQ-SF) (32) will be used to assess the frequency, duration, and intensity of physical activity before and after the 100-day intervention.

### Permitted concomitant care

The consumption of probiotics or herbal medicines is prohibited during this trial due to potential drug interactions. Participants are permitted to use analgesia if needed during the trial. If participants are recommended by their usual healthcare practitioners to start any new medications during the trial, they will be encouraged to discuss this with the PI to determine whether this affects their trial eligibility. Participants will be asked to report all medication use at Days 0, 30, 50, and 100.

### Adverse events

Complications will be reported and described by duration, severity, outcome, treatment, and relation to study treatment or cause. Information on experienced side effects will be collected on Days 30, 50, and 100. Participants are encouraged to discuss any experienced side effects with the PI and their General Practitioner. All serious adverse events will be reported to the ethics committee. An independent data safety monitoring committee consisting of a senior researcher, clinician, and statistician will be formed to review all adverse events and judge their severity and relatedness to the trial using established standards (33). The committee will inform the PI if the adverse events warrant terminating the study. All participants will be immediately informed of the termination. The trial sponsor may also wish to request for early termination of the study.

In the event of injury during this trial, participants are eligible to apply for compensation from the New Zealand Crown entity, Accident Compensation Corporation. It is made clear in the Participant Information Sheet that claims are not automatically accepted.

### Data management

A secure database will be generated, which will be password-protected on a university-provided server and accessible only to research team members. Once written informed consent is obtained from eligible participants, a unique study ID code will be allocated. From this point, all study data and samples will be link-anonymised. All results from study assessments will be entered into password-protected electronic case report forms labeled with participants' unique study ID codes. Paper forms of study data collection will be stored in locked cabinets.

All study data will be collected online through the WeChat social media platform (owned by Tencent Inc.), using an official account and mini-programme specifically designed for this trial by the National Institute for Health Innovation (NIHI), University of Auckland, New Zealand. The decision to use WeChat was based on a prior WeChat-based intervention conducted in Auckland, New Zealand, which targeted a similar population (34, 35). Participants who own a smartphone or tablet and have a WeChat account will have access to the official account and mini-programme of the study. The content, user interface, and user experience design of the official account and the mini-programme were developed based on standardized questionnaires (29–32) and by following the WeChat mini-programme design guidelines (36). All questionnaires will be delivered *via* the WeChat platform. Data will be recorded and stored in the mini-programme. At the end of the study, data will be exported from the mini-programme for analysis. Exported data will be stored safely and password protected. Data in the mini-programme will be purged after 10 years. No routine audits of trial conduct are planned.

### Participant adherence

Links to questionnaires and reminders to complete questionnaires will be provided *via* WeChat. Questionnaires can be completed online at any convenient time, which is expected to promote data completeness. If participants have not completed the required questionnaires within 7 days, a research assistant will phone them and provide an opportunity to complete these questionnaires *via* the telephone. Research assistants will also check responses once questionnaires have been returned and will contact participants to provide any missing data, if necessary. Participants will self-report daily treatment adherence by scanning the unique QR code on the chocolate wrapper using the WeChat mini-programme. Participants will also be asked to retain all chocolate wrappers (opened and unopened) and return them at the final visit (Day 100).

Participants who discontinue treatment early will remain in the trial with ongoing data collection. This includes those who no longer meet the eligibility criteria during the trial due to a change in medication or medical history. If participants withdraw from the study, consent will be sought to use all data collected up to the time of study withdrawal. They will not be replaced.

### Statistical methods

The primary outcomes are change in insulin resistance as defined by HOMA and patient-reported joint pain as measured using WOMAC. A sample size of 150 participants (75 per arm) will provide 90% power at an overall significance level of 5% (two-sided) to detect a standardized effect size of 0.625 on either of the two co-primary outcomes, with the Bonferroni correction (α = 0.025 for each outcome), allowing for 10% loss to follow-up. This effect size is equivalent to a group difference of 1 point on patient-reported joint pain *via* WOMAC and a 0.5-unit difference in change in HOMA, both of which are considered clinically significant. Standard deviation (SD) calculations for pain reduction and change in HOMA were based on previous trials (37, 38).

Statistical analysis will be performed at the end of the trial following an intention-to-treat (ITT) principle, including all randomized participants in the group they were allocated to. No interim analyses are planned during the trial. Trial data will be exported to SAS version 9.4 (SAS Institute Inc., Cary, USA) for analysis. All statistical tests will be two-sided at a 5% significance level. A statistical analysis plan will be prepared a priori by the trial statistician to guide the final data analysis.

Baseline demographics and clinical characteristics of all trial participants will be summarized using descriptive statistics by treatment group. Continuous variables will be presented as mean, SD, median, and interquartile range, as appropriate. Categorical variables will be presented as frequencies and percentages. No formal statistical tests will be conducted at baseline, as previously recommended for parallel-group randomized trials (39).

Outcomes measured at the end of the intervention will be summarized descriptively by the treatment group, together with their baseline measures. Adverse events will be summarized separately. A generalized linear regression model will be used to estimate the effect of the intervention on each outcome, using a link function appropriate to the distribution of the outcome variable. The model will adjust for baseline outcome value and important baseline confounders as pre-specified. Missing data on the primary outcomes will be imputed using multiple imputations in the main ITT analysis and explored in sensitivity analysis under different assumptions to test the robustness of the main trial results. Per-protocol analysis will include participants adherent to the treatment without major protocol deviations. No data imputation will be considered on secondary outcomes. The CONSORT 2010 statement will be used as the guidelines to report the main trial results.

### Communicating protocol amendments

Amendments to the protocol or any other supporting documents which may directly affect the participants in the trial, the scientific value of the trial, the conduct or management of the trial, or the quality or safety of the trial will be first communicated to the Health and Disability Ethics Committee. Participants will be informed of all amendments which impact them. If needed, additional consent will be requested and registered. Non-substantial amendments will be recorded and filed. Online trial registries will be updated accordingly.

### Data ownership

Individual trial data will remain the property of the individual participants. The PI, co-investigators, and trial sponsor will have access to the final non-identifiable trial dataset at the end of the study. Information from this trial may lead to the development of a commercial product. Project intellectual property (IP) will be owned by AUT and managed by its commercialization and technology transfer office, AUT Ventures. AUT Ventures will meet every 6 months with the project team during the latter half of the project to evaluate opportunities for IP protection. Expected areas of IP protection include the exact formulation and production method of the new product. AUT Ventures will exclusively license the product formulation to the commercial partner, Beyond Capital MedTech Management Limited, on a royalty-free basis for its primary market of Asia. Participants and their families will not receive any financial benefits or compensation, nor have any rights in any developments, inventions, or other discoveries which may arise from the collected information.

## Discussion

Chronic musculoskeletal joint pain and type 2 diabetes are significant contributors to the global burden of disease. Although pharmaceutical drugs are beneficial, interest in complementary therapies is growing. Green-lipped mussel and fucoidan extracts already exist as separate supplements and are currently being used for their anti-inflammatory properties. However, to our knowledge, the benefits of combining green-lipped mussel and fucoidan powders have never been explored. Proposed therapeutic benefits of the mussel-fucoidan supplement include reduced joint pain levels, decreased dependence on analgesic medication, and improved glucose uptake. This trial will provide greater insight into mussel-fucoidan combinations and help develop high-value foods for health. Extracting fucoidan from *U. pinnatifida* also has the potential to add commercial value to the mussel industry as it is currently considered an unwanted waste product.

We are exclusively recruiting Chinese participants, thus our findings may not be generalisable to the global population as differences in habitual diets and metabolism (40) may affect the bioavailability of the mussel-fucoidan supplement. However, this trial could be later extended to include other ethnicities. Another limitation is the use of self-reported questionnaires, which have been translated into Mandarin but are not validated for use in our target population (P4, MSK-HQ, NZPAQ-SF). Existing questionnaires translated into Mandarin and validated in a Chinese population are designed for patients with knee osteoarthritis (41) or rheumatoid arthritis (42) and thus were not suitable for this trial. Another limitation is that pharmacokinetic study of fucoidan was not planned, which could be essential for determining the optimal dose in the future. Although fucoidan is a substance with complicated composition, biomarker approach used in previous *in vivo* study could be used to assess the pharmacokinetic of fucoidan (43).

Overall, this trial will provide novel data on the potential utility of a mussel-fucoidan supplement in reducing joint pain and insulin resistance and may provide an alternative to current analgesic and hypoglycaemic medications.

### Trial status

The protocol version number is 12 and date is 1 February 2022. The recruitment began on 8 February 2022, and the approximate date recruitment will be completed is 30 September 2022.

## Strengths and limitations of this study

- This study is the first randomized, double-blinded, placebo-controlled trial to combine green-lipped mussel powder with fucoidan extract for the treatment of joint pain and insulin resistance in prediabetes
- This is the first trial investigating the effect of green-lipped mussel powder in a Chinese population
- Its exclusive recruitment of Chinese participants living in New Zealand may limit generalisability of this trial to other ethnic groups
- As a two-arm design this trial does not elucidate the effects of combining mussel with fucoidan.

## Ethics statement

The studies involving human participants were reviewed and approved by the Health and Disability Ethics Committee (number: 20/STH/153). The patients/participants provided their written informed consent to participate in this study.

## Author contributions

JL is the trials PI who has supervised and overseen all trial procedures and documentation. JL co-leads this trial with RM, who assisted with trial design, and will contribute to participant recruitment and clinical assessments. NS and DO'B provided practical clinical expertise and assisted with trial design. They will conduct clinical assessments and contribute to data analysis and interpretation. YJ and JC contributed to trial design and developed the statistical analysis plan. YJ will lead the final statistical analysis. AT led the development of the trial protocol and wrote this manuscript. All authors contributed to the article and approved the submitted version.

## Funding

This work was supported by the High-Value Nutrition National Science Challenge (HVN 3719491 1905), Beyond Capital MedTech Management Limited, and Education New Zealand (NZ-China Tripartite Research Collaboration Fund).

## Conflict of interest

The authors declare that the research was conducted in the absence of any commercial or financial relationships that could be construed as a potential conflict of interest.

## Publisher's note

All claims expressed in this article are solely those of the authors and do not necessarily represent those of their affiliated organizations, or those of the publisher, the editors and the reviewers. Any product that may be evaluated in this article, or claim that may be made by its manufacturer, is not guaranteed or endorsed by the publisher.
